# Using a Novel Partitivirus in *Pseudogymnoascus destructans* to Understand the Epidemiology of White-Nose Syndrome

**DOI:** 10.1371/journal.ppat.1006076

**Published:** 2016-12-27

**Authors:** Vaskar Thapa, Gregory G. Turner, Susan Hafenstein, Barrie E. Overton, Karen J. Vanderwolf, Marilyn J. Roossinck

**Affiliations:** 1 Department of Plant Pathology and Environmental Microbiology, Center for Infectious Disease Dynamics, Pennsylvania State University, University Park, PA, United States of America; 2 Pennsylvania Game Commission, Harrisburg, PA, United States of America; 3 Department of Microbiology, Pennsylvania State University College of Medicine, Hershey, PA, United States of America; 4 Department of Biology, Lock Haven University of Pennsylvania, Lock Haven, PA, United States of America; 5 New Brunswick Museum, Saint John, NB, Canada; Division of Clinical Research, UNITED STATES

## Abstract

White-nose syndrome is one of the most lethal wildlife diseases, killing over 5 million North American bats since it was first reported in 2006. The causal agent of the disease is a psychrophilic filamentous fungus, *Pseudogymnoascus destructans*. The fungus is widely distributed in North America and Europe and has recently been found in some parts of Asia, but interestingly, no mass mortality is observed in European or Asian bats. Here we report a novel double-stranded RNA virus found in North American isolates of the fungus and show that the virus can be used as a tool to study the epidemiology of White-nose syndrome. The virus, termed *Pseudogymnoascus destructans* partitivirus-pa, contains 2 genomic segments, dsRNA 1 and dsRNA 2 of 1.76 kbp and 1.59 kbp respectively, each possessing a single open reading frame, and forms isometric particles approximately 30 nm in diameter, characteristic of the genus *Gammapartitivirus* in the family *Partitiviridae*. Phylogenetic analysis revealed that the virus is closely related to *Penicillium stoloniferum virus S*. We were able to cure *P*. *destructans* of the virus by treating fungal cultures with polyethylene glycol. Examination of 62 isolates of *P*. *destructans* including 35 from United States, 10 from Canada and 17 from Europe showed virus infection only in North American isolates of the fungus. Bayesian phylogenetic analysis using nucleotide sequences of the viral coat protein geographically clustered North American isolates indicating fungal spread followed by local adaptation of *P*. *destructans* in different regions of the United States and Canada. This is the first demonstration that a mycovirus potentially can be used to study fungal disease epidemiology.

## Author Summary

Many species of bats in North America have been severely impacted by a fungal disease, white-nose syndrome, that has killed over 5 million bats since it was first identified in 2006. The fungus is believed to have been introduced into a cave in New York where bats hibernate, and has now spread to 29 states and 4 Canadian provinces. The fungus is nearly identical from all sites where it has been isolated; however, we discovered that the fungus harbors a virus, and the virus varies enough to be able to use it to understand how the fungus has been spreading. This study used samples from infected bats throughout Pennsylvania and New York, and New Brunswick, Canada, as well a few isolates from other northeastern states. The evolution of the virus recapitulates the spread of the virus across these geographical areas, and should be useful for studying the further spread of the fungus.

## Introduction

*Pseudogymnoascus destructans* (Pd; previously named *Geomyces destructans*) is an emerging fungal pathogen responsible for a fatal disease, white-nose syndrome (WNS) in hibernating bats in North America [1–3]. Experts estimate over 5 millions bats died from WNS in North America since the disease was first noted in New York in 2006 [4–6]. Currently WNS has spread to at least 29 states in the United States (plus three additional states where Pd presence has been confirmed, but not WNS) and five provinces in Canada [4]. The fungus is widely distributed in Europe [6, 7] and recently has been reported from the northeast of China and Siberia [8, 9], but no mass mortality has been reported in European or Asian bats [6, 8]. Pd’s lethal effect on North American bats coupled with its clonal genotype in North American isolates [10, 11], its single mating type [12] and the absence of close relatives [13] led many researchers to hypothesize a recent introduction to North America [1, 6, 14, 15]. Pd is confirmed in seven North American [1, 4] and 13 European species of bats [4, 9]. The natural history of the genus *Pseudogymnoascus* and its allies indicate that they are commonly isolated from soils in colder regions of the world [16]. Unlike Pd many of its close relatives are cellulolytic saprobes and non-pathogenic [16, 17].

Mycoviruses associated with fungi have drawn interest because of their potential roles in fungal biology and pathogenicity [18]. Mycoviruses are very frequent in fungi and generally maintain a persistent lifestyle [19]. Horizontal transmission is very rare, and is likely restricted to closely related strains, although phylogenetic studies indicate transmission among species has occurred [20]. Transmission has only been documented in a few cases outside the laboratory [21]. Most mycoviruses are cryptic with no known biological effects on their fungal hosts, although there is a lack of research in this area. However, there are significant examples where mycoviruses play important roles in fungal biology and ecology [22]. Here we used mycoviruses of Pd as a tool to study the epidemiology of WNS. We investigated mycoviruses in Pd and show that population variation of a Pd-mycovirus can be useful in tracing the spread of WNS.

## Results

### A partitivirus infection in North American isolates of Pd

**Fig 1:**
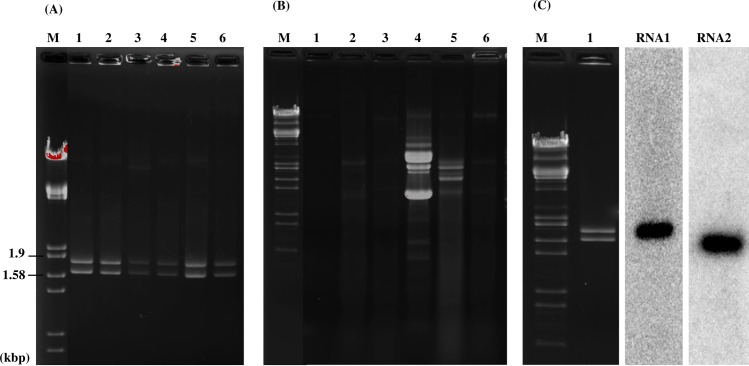
Agarose gel electrophoresis and northern blot analysis of PdPV-pa genomic RNA. (A) dsRNA profiles of six representative isolates of Pd from North America; 1 = LB-01, 2 = TC-01, 3 = 20631–21, 4 = M3906, 5 = 461202 and 6 = 421101, showing characteristic bands for PdPV. Descriptions of isolates are in Table 1. (B) dsRNA profiles of 6 representative isolates of Pd from Europe; 1 = CMF-2583, 2 = CCF-3937, 3 = CCF-4125, 4 = CCF-4127, 5 = CCF-4128 and 6 = CCF-4129, showing no detectable dsRNA (lanes 1, 2, 3, and 6) or a different pattern from PdPV-pa (lanes 4 and 5). Descriptions of isolates are in Table 1. (C) Northern blot analyses of PdPV-pa RNA probed for RNA 1 and RNA 2 as marked. M is a size marker in all panels (lambda DNA digested with *Eco*RI and *Hind*III

We examined 62 isolates of Pd from North American and European bats for mycoviruses (Table 1). The isolates were cultured from four North American and one European species of bats and were collected from 2008 to 2015. North American isolates included 14 from Pennsylvania, seven from New York, six from West Virginia, three from North Carolina, three from Vermont, one from Ohio, one from Indiana and 10 from New Brunswick, Canada. We screened 16 isolates of Pd from the Czech Republic and one isolate from Slovakia in Europe. Double-stranded RNA (dsRNA) extracted from all North American isolates showed two bands—a larger band close to 1.8 kb (RNA 1) and a smaller band close to 1.6 kb (RNA 2) in electrophoretic analysis (Fig 1A). None of the European isolates contained these dsRNAs, although two, CCF-4127 and CCF-4128, had dsRNAs profiles different from that of the North American isolates (Fig 1B). We found no dsRNAs of viral origin in five isolates of *Geomyces* sp. from Antarctic soil or in six isolates of *Pseudogymnoascus* sp. from cave soils in Pennsylvania (S1 Table). The dsRNA enrichment method is based on the premise that uninfected plants or fungi normally do not contain detectable amounts of high molecular weight dsRNA, and, when present, dsRNA is an indicator of a viral genome [23]. Sanger sequencing of cDNA clones from RNAs 1 and 2 of the North American isolates of Pd obtained from random primed RT-PCR provided nearly complete genomic sequences; ends were determined by 5'- primer ligated RNA ligase mediated-rapid amplification of cDNA ends (RLM-RACE) [24] providing consensus genomic sequences for RNAs 1 and 2 of 1761 bp and 1590 bp. Northern-blots using cDNA clones from RNA 1 or RNA 2 as probes confirmed the identity of the dsRNA bands (Fig 1C). We named this new virus *Pseudogymnoascus destructans* partitivirus-pa (PdPV-pa; the pa indicates the sequenced isolate is from Pennsylvania).

**Table 1:** Attributes of *Pseudogymnoascus destructans* isolates used in the study

Isolate ID^1^	Location	Date collected^2^	Host species	Source^3^	PdPV-pa^4^
**LB-01**	Blossburg Mine, Tioga county, PA, USA	03/22/2011	*Myotis lucifugus*^5^	RL	+
**LB-02**	Kennerdell, PA, USA	03/13/2012	*Myotis lucifugus*	RL	+
**LB-03**	Indian Cave, Somerset Co, PA, USA	02/16/2013	*Myotis lucifugus*	RL	+
**LB-04**	Centre Co, PA, USA	03/25/2012	*Myotis lucifugus*	RL	+
**LB-05**	Centre Co, PA, USA	03/28/2012	*Myotis lucifugus*	RL	+
**LB-06**	Cook Forest State Park, Cooksburg, PA, USA	03/21/2012	*Myotis lucifugus*	RL	+
**LB-07**	Cook Forest State Park, Cooksburg, PA, USA	03/21/2012	*Myotis lucifugus*	RL	+
**LB-08**	Cook Forest State Park, Cooksburg, PA, USA	03/21/2012	*Myotis lucifugus*	RL	+
**LB-B**	Blosssburg Mine, Tioga County, PA, USA	03/22/2011	*Myotis lucifugus*	RL	+
**LB-55571^6^**	Canoe Creek, Hollidaysburg, PA, USA	04/09/2014	*Myotis lucifugus*	RL	+
**LB-55617^6^**	Canoe Creek, Hollidaysburg, PA, USA	04/24/2014	*Myotis lucifugus*	RL	+
**BB-06^7^**	Layton Fire Clay Mine, Allegheny Co, PA, USA	03/04/2015	*Eptesicus fuscus*^8^	RL	+
**BB-10^7^**	Layton Fire Clay Mine, Allegheny Co, PA, USA	03/04/2015	*Eptesicus fuscus*	RL	+
**NLE-01VT**	Plymouth Cave, Plymouth, VT, USA	03/26/2015	*Myotis septentrionalis*^9^	RL	+
**LB-01IN**	Wyandotte Cave, Leavenworth, IN, USA	04/20/2015	*Myotis lucifugus*	RL^10^	+
**TC-01**	Blossburg Mine, Tioga County, PA, USA	03/22/2011	*Perimyotis subflavus*^11^	RL	+
**20631–21^12^**	Williams Hotel, NY, USA	2008	*Myotis lucifugus*	CFMR	+
**M3902**	WV, USA	02/23/2010	*Myotis lucifugus*	CFMR	+
**M3903**	WV, USA	03/12/2010	*Perimyotis subflavus*	CFMR	+
**M3905**	NC, USA	02/03/2011	*Myotis lucifugus*	CFMR	+
**M3906**	NC, USA	02/03/2011	*Perimyotis subflavus*	CFMR	+
**M3907**	WV, USA	03/23/2011	*Myotis lucifugus*	CFMR	+
**M3908**	NC, USA	02/08/2011	*Myotis lucifugus*	CFMR	+
**M3909**	OH, USA	03/22/2011	*Myotis lucifugus*	CFMR	+
**M3910**	WV, USA	03/23/2011	*Myotis lucifugus*	CFMR	+
**M3911**	WV, USA	03/11/2011	*Perimyotis subflavus*	CFMR	+
**M3912**	WV, USA	03/23/2011	*Myotis lucifugus*	CFMR	+
**M2443**	NY, USA	04/13/2010	*Perimyotis subflavus*	CFMR	+
**M2461**	NY, USA	05/11/2010	*Myotis lucifugus*	CFMR	+
**M2332**	Dannemora, Clinton, NY, USA	03/11/2009	*Myotis lucifugus*	CFMR	+
**M2333**	Dannemora, Clinton, NY, USA	03/11/2009	*Myotis lucifugus*	CFMR	+
**M2334**	Newstead, Erie, NY, USA	03/12/2009	*Myotis lucifugus*	CFMR	+
**M2335**	Ithaca, Tompkins, NY, USA	03/16/2009	*Myotis lucifugus*	CFMR	+
**M4513**	VT, USA	_	*Myotis lucifugus*	CFMR	+
**M4514**	VT, USA	_	*Myotis lucifugus*	CFMR	+
**461202^13^**	Glebe Mine, New Brunswick, Canada	2012	*Perimyotis subflavus*	NBM	+
**681102^13^**	Glebe Mine, New Brunswick, Canada	2013	*Perimyotis subflavus*	NBM	+
**671105^13^**	Glebe Mine, New Brunswick, Canada	2013	*Perimyotis subflavus*	NBM	+
**92203^14^**	White Cave, New Brunswick, Canada	2012	*Myotis lucifugus*	NBM	+
**21201^13^**	White Cave, New Brunswick, Canada	2012	*Myotis lucifugus*	NBM	+
**82205^14^**	White Cave, New Brunswick, Canada	2012	*Myotis lucifugus*	NBM	+
**642103^14^**	Berryton Cave, New Brunswick, Canada	2012	*Myotis lucifugus*	NBM	+
**212104^14^**	Markhamville Mine, New Brunswick, Canada	2012	*Myotis septentrionalis*	NBM	+
**421101^13^**	Harbell Cave, New Brunswick, Canada	2012	*Myotis septentrionalis*	NBM	+
**702107^14^**	Markhamville Mine, New Brunswick, Canada	2013	*Perimyotis subflavus*	NBM	+
**CMF-2498**	Harmanecka Cave, Slovakia, Europe	2013	*Myotis myotis*^15^	CFMR^16^	-
**CMF-2583**	Na Pomezi Caves, Moravia, Czech Republic, Europe	2013	*Myotis myotis*	CFMR^16^	-
**CMF-2584**	Na Pomezi Caves, Moravia, Czech Republic, Europe	2013	*Myotis myotis*	CFMR^16^	-
**CCF-3937**	Mala Amerika, Bohemian Karst, Czech Republic, Europe	2010	*Myotis myotis*	CFMR^16^	-
**CCF-3938**	Solenice, Czech Republic, Europe	2010	*Myotis myotis*	CFMR^16^	-
**CCF-3941**	Mala Amerika, Bohemian Karst, Czech Republic, Europe	2010	*Myotis myotis*	CFMR^16^	-
**CCF-4103**	Herlikovice, Czech Republic, Europe	2011	*Myotis myotis*	CFMR^16^	-
**CCF-4125**	Homi Alberice, Czech Republic, Europe	2011	*Myotis myotis*	CFMR^16^	-
**CCF-4127**	Herlikovice, Czech Republic, Europe	2011	*Myotis myotis*	CFMR^16^	-
**CCF-4128**	Herlikovice, Czech Republic, Europe	2011	*Myotis myotis*	CFMR^16^	-
**CCF-4129**	Pistov, Czech Republic, Europe	2011	*Myotis myotis*	CFMR^16^	-
**CCF-4130**	Fucna-Otov, Czech Republic, Europe	2011	*Myotis myotis*	CFMR^16^	-
**CCF-4131**	Vyskov, Czech Republic, Europe	2011	*Myotis myotis*	CFMR^16^	-
**CCF-4132**	Pernink, Czech Republic, Europe	2011	*Myotis myotis*	CFMR^16^	-
**CCF-4247**	Morina, Czech Republic, Europe	2012	*Myotis myotis*	CFMR^16^	-
**CCF-4350**	Mala Amerika, Bohemian Karst, Czech Republic, Europe	2012	*Myotis myotis*	CFMR^16^	-
**CCF-4351**	Mala Amerika, Bohemian Karst, Czech Republic, Europe	2012	*Myotis myotis*	CFMR^16^	-

^1^ All isolates were collected as bat wing samples except as indicated. Isolate numbers are reference numbers for individual collections (Source)

^2^ Collection dates where known.

^3^ +, virus positive by dsRNA analysis and RT-PCR; -, virus negative

^4^ common name: little brown bat

^5^ RL, Roossinck Lab Collection at Penn State, culture substrates collected by the Pennsylvania Game Commission except as indicated; CFMR, Reference Culture Collection at the Center for Forest Mycology Research (http://www.fpl.fs.fed.us/research/centers/mycology/culture-collection.shtml); NBM, New Brunswick Museum (http://www.nbm-mnb.ca)

^6^ collected as wing swab

^7^ collected as wing punch

^8^ common name big brown bat

^9^ common name northern long-eared bat

^10^ Culture substrate was collected by Lori Pruitt, United States Fish and Wildlife Service, Bloomington Office, IN

^11^ common name eastern pipistrelle or tri-colored bat

^12^ 20631–21 has American Type Culture Collection identifier MYA-4855

^13^ swab from the bat wing and muzzle skin

^14^ swab from the bat dorsal fur

^15^ common name, greater mouse-eared bat

^16^ Originally from Dr. Miroslav Kolarik, Laboratory of Fungal Genetics and Metabolism, Czech Republic, but held in CFMR collection

A BLASTx search of GenBank showed closest similarity of RNA 1 of PdPV-pa with RNA 1 of *Penicillium stoloniferum virus* S (PsV-S), with 76% amino acid (aa) identity. Similarly, RNA 2 of Pd showed closest similarity with the RNA 2 of PsV-S with 67% aa identity. PsV-S is the type species of the genus *Gammapartitivirus* in the family *Partitiviridae* [25].

### Characterization and phylogeny of PdPV-pa

**Fig 2:**
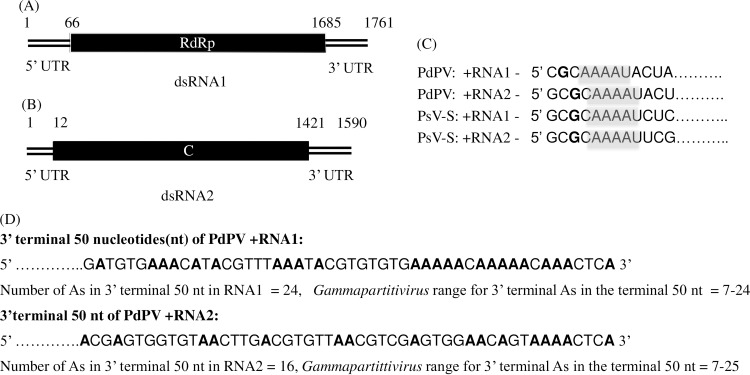
Genome organization and conserved features in RNA 1 and RNA 2 of PdPV-pa. (A) PdPV-pa genomic dsRNA 1 with a single open reading frame (ORF) (nt 66–1685) coding for a putative RdRp. (B) PdPV genomic dsRNA 2 with a single ORF (nt 12–1421) coding for a putative CP. (C) 5' + strand termini of PdPV-pa and *Penicillium stoloniferum virus-S* (PsV-S) (type species of the *Gammapartitivirus* genus) with conserved GCAAAA sequence where the nucleotides following G in the next 5 or 6 positions are either C, A or U, but not G. (D) 3' terminal 50 nucleotide sequence of RNAs 1 and 2 of PdPV-pa that is rich in A residues typical of the genus *Gammapartitivirus*.

**Fig 3:**
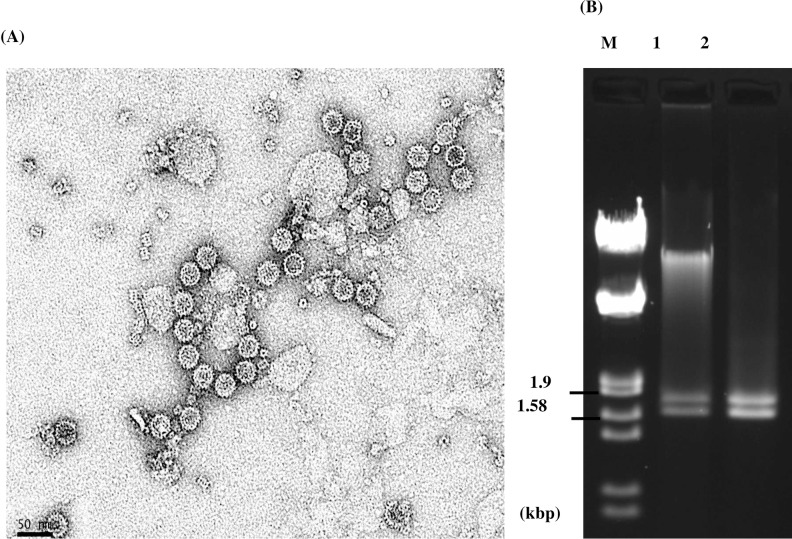
Virus particle morphology and packaged genomic dsRNAs of PdPV-pa. (A) Particles purified from Pd isolate BB-06, were examined by TEM after negative staining with uranyl formate. The bar indicates 50 nm. (B) Agarose gel electrophoresis profile of PdPV-pa genomic dsRNA segments (lane 1) isolated from the purified virus preparation and the dsRNA segments (lane 2) extracted from mycelia of the same Pd isolate.

Sequence analysis of RNA 1 of PdPV-pa predicted a single open reading frame (ORF) of 540 aa (60 kDa) that codes for a putative RNA-dependent RNA polymerase (RdRp) (Fig 2A). RNA 2 also contained a single ORF of 470 aa (52 kDa) that codes for a putative coat protein (CP) (Fig 2B). Amino acid level sequence identity of PdPV-pa RdRp and CP with the approved members of genus *Gammapartitivirus* in the family *Partitiviridae* ranges from 58% - 76% and 36% - 67% respectively, which are within the species demarcation criteria (RdRp ≤ 90%; CP ≤ 80%) of the genus [42]. Further, the 5' termini of PdPV-pa RNAs 1 and 2 coding strand share a conserved C**G**CAAAA… sequence, where **G** is followed by A, U, or C but not G in the next 5 to 6 nucleotide positions, characteristic of the genus *Gammmapartitivirus* [25] (Fig 2C). Similarly, the 3' terminal 50 nucleotides of RNAs 1 and 2 were adenosine (A) rich in the range (7–24 nt) typical of members of the *Gammapartitivirus* genus [25] (Fig 2D). PdPV-pa particles were purified from mycelia of Pd and negative-stain transmission electron microscopy showed isometric particles of approximately 30 nm diameter, characteristic of members of the *Partitiviridae* (Fig 3A). PdPV-pa dsRNAs were also extracted from the purified virus particles to reconfirm their presence as genomic RNAs (Fig 3B).

**Fig 4:**
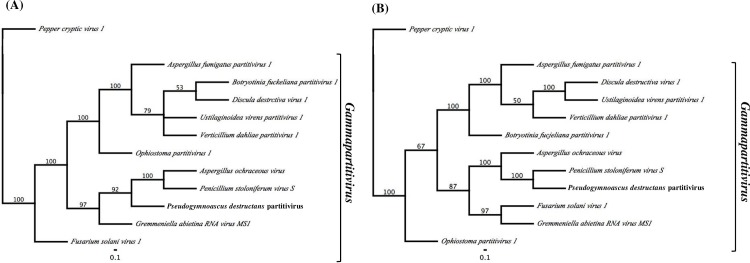
Phylogenetic analysis of PdPV-pa. Bayesian trees constructed aa sequences of PdPV-pa (shown in **bold)** and *Gammapartitivirus* sequences available online from GenBank (S1 Table). The numbers at nodes in both trees represented posterior probability support. *Pepper cryptic virus 1*, type member of genus *Deltapartitivirus* of *Partitiviridae* family was used as the outgroup. Branches with posterior probability support <50% were collapsed. (A) RdRp tree (B) CP tree.

Bayesian trees constructed using aa sequences from the RdRp and CP of PdPV-pa clustered PdPV-pa with other members of genus *Gammapartitivirus* in the *Partitiviridae* family (Fig 4A & 4B). In both RdRp and CP trees, PdPV-pa appeared as a sister branch to PsV-S with strong posterior probability support of 92% and 100% respectively suggesting PdPV-pa is evolutionary close to PsV-S. The genome structure of PdPV-pa, conserved features in its RNAs explained above, its particle morphology, its RdRp and CP amino acid sequence identity within species demarcation criteria, and phylogenetic analyses all confirmed that PdPV-pa is a novel partitivirus belonging to genus *Gammapartitivirus* in the family *Partitiviridae*.

### Curing of *Pseudogymnoascus destructans*

**Fig 5:**
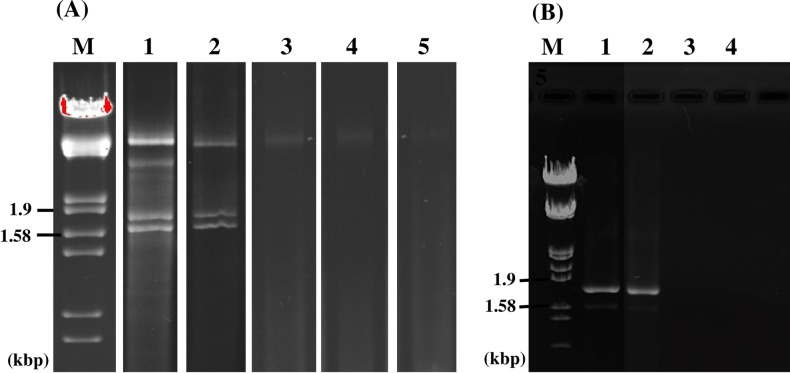
Curing Pd of PdPV-pa by treating with PEG-induced matric stress on water availability. (A) Profiles of dsRNA extracted from Pd cultures treated with 0 MPa (lane 1 as control), -1 MPa (lane 2), -2 MPa (lane 3), -3 MPa (lane 4) and -4 MPa (lane 5) induced by PEG. Note absence dsRNAs corresponding to PdPV at -2 MPa, -3 MPa and -4 MPa. (B) RT-PCR using dsRNAs extracted from the different PEG induced treatments described above with PdPV-pa specific RdRp primers. The lane numbers corresponds to the matric potential order as in (A).

We attempted several methods including single spore isolation, hyphal tip culture, protoplast culture, heat therapy and nutritional and chemical stress that involved application of the antiviral drugs cycloheximide or ribavirin, to cure Pd of the PdPV-pa infection. However, only partial success was achieved with high concentrations of cycloheximide (25 μg/ml) and ribavirin (300 μM) treatments after three passages. PdPV-pa remained suppressed in the fungus treated with cycloheximide or ribavirin when grown in media with the drug but once the fungus was transferred to drug-free media the virus reappeared. Finally, our attempt to cure the fungus using polyethylene glycol (PEG)-induced matric potential in minimal nutrition media made PdPV-pa undetectable. PdPV-pa infection in Pd was checked under matric potential gradients starting from -2MPa, -3MPa to -4MPa. We did not observe visible germination of Pd conidia or mycelia growth at -5MPa and -6MPa. PdPV-pa was undetected in PEG treated Pd isolates when evaluated by dsRNA extraction and RT-PCR with RdRp specific primers for PdPV-pa (Fig 5A & 5B). The detection limit of PdPV-pa in Pd was determined to be approximately 380 copies per cell (S1 Appendix). We enriched the viral dsRNA from total nucleic acid extracted from a defined number of Pd conidia followed by measurement of dsRNA concentration, and serial dilutions to determine the end-point of detection.

**Fig 6:**
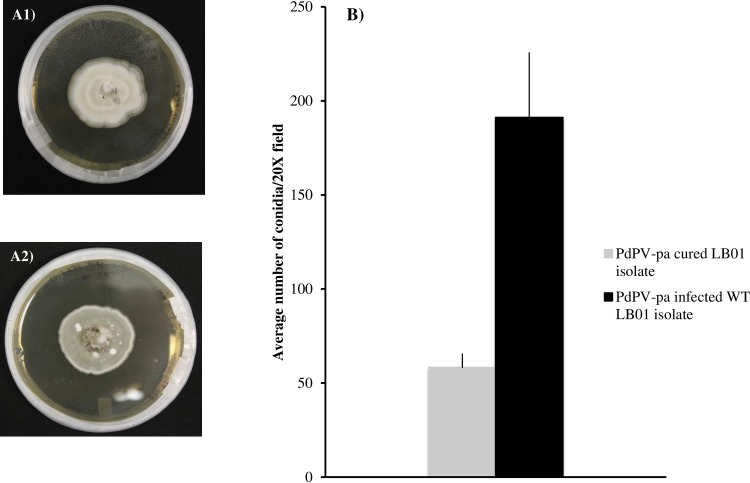
Changes in Pd after virus curing. A) white colony of LB01 isolate of Pd grown in 0.5X Sabouraud dextrose agar (SDA) media after treatment with PEG lowered matric potential media where PdPV-pa was undetected, and wild type LB01 isolate of Pd grown in SDA media showing characteristic gray pigmentation with PdPV-pa infection. Both cultures were grown for three weeks in the dark at 10°C. B). Conidia were collected from equal amounts of mycelial mass from PdPV-pa cured and infected isolates and suspended in 200 μl of sterile water and then diluted 10X before viewing under a microscope. Bars show the average number of conidia per 20X field with error bars calculated from 20 replicates, each from PdPV-pa cured and infected samples. The difference is statistically significant at α = 0.05.

Pd isolates where PdPV-pa was undetected after PEG treatment lost the characteristic gray pigmentation of wild type Pd and appeared white (Fig 6A). The virus-free isolate also produced significantly less conidia in comparison to wild type isolate (Fig 6B).

Although PEG treatments were successful in obtaining a PdPV-pa free isolate of Pd, PdPV-pa tolerance to many other stresses mentioned above indicate that PdPV-pa is tightly associated with the Pd isolates from North America.

### Genetic variability in the North American population of PdPV-pa

**Fig 7:**
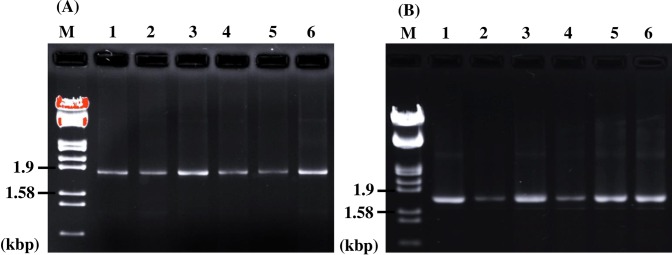
RT-PCR of North American isolates of *Pseudogymnoascus destructans* partitivirus-pa (PdPV-pa) using RNA-dependent RNA polymerase (RdRp) and coat protein (CP) specific primers. Agarose gel electrophoresis of amplicons of North American isolates of PdPV-pa, amplified by RT-PCR with RdRp specific primers (A) or CP specific primers (B). Lanes 1–6 in both gel images are different isolates and M is the marker lane as described in Fig 1

Genetic variability of the RdRp and CP regions was analyzed in 45 North American isolates of PdPV-pa by amplification using specific primers followed by sequence analysis (Fig 7A & 7B). Using a 930 bp region of RdRp amplicons after trimming and alignment, we found the average percentage identity ranged from 99.7 to 99.9 among the 45 isolates. The high level of conservation in the RdRp is also reflected by a total of only 15 segregating sites, including seven singletons among the isolates examined. For the CP, nucleotide variability was higher: in a 1088 bp of amplicon of the CP, the average percent identity ranged from 96.8 to 98.4 and included 127 segregating sites out of which 69 were singletons.

### Phylogenetic relationships of PdPV-pa among North American isolates

**Fig 8:**
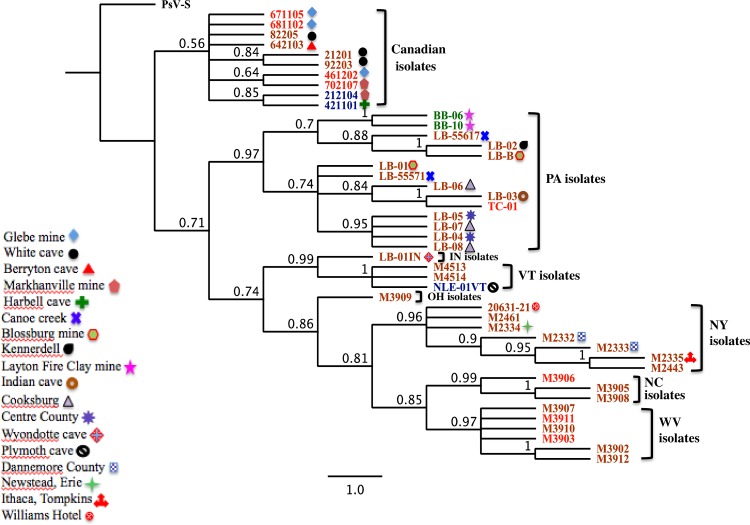
Phylogenetic analysis of North American isolates of PdPV-pa. Rooted Bayesian trees constructed from the nucleotide sequences of the CP amplicon from 45 North American PdPV-pa isolates, as described in the Materials and Methods. *Penicillium stolonoferum virus*-S coat protein sequence was used as the outgroup. The numbers in nodes refer to posterior probability values. The isolate IDs were color coded for bat species, brown for *Myotis lucifugus* (little brown bat), red for *Perimyotis subflavus* (tri-colored bat) blue for *Myotis septentrionalis* (northern long eared bat) and green for *Eptesicus fuscus* (big brown bat). Different shapes associated with each isolate refer to the specific location of the collection. The scale value represents nucleotide substitutions per site. Refer to Table 1. for the isolate details.

The Bayesian tree based on the RdRp nucleotide sequences of 45 North American isolates of PdPV-pa produced a largely unresolved tree with no clusters with significant support. However, the Bayesian tree constructed from the nucleotide sequences of the CP clustered the 45 PdPV-pa isolates into two major clades based on their geographical distribution (Fig 8). One clade was comprised of Canadian isolates; the other clade included isolates from the USA, although the posterior probability of this separation was lower than for other branching in the tree. The USA clade further included well supported clusters of isolates from New York, Pennsylvania, West Virginia, North Carolina, Vermont, Indiana and Ohio. Indiana and Ohio had one isolate each and separated as sister branches. The separate topologies of USA and Canadian clusters indicate independent diversification of Pd isolates subsequent to movement to particular regions. Within each major clade there were examples of sub-branching topologies representing isolates based on their local distribution although the pattern was not consistent throughout. The phylogeny of the PdPV-pa isolates showed no structure based on the taxonomy of the bats indicating that Pd is a generalist pathogen that is transmitted readily across bat species.

## Discussion

In this study, we isolated and characterized a novel virus, PdPV-pa, from the pathogenic filamentous fungus causing WNS in North American bats. Based on the nucleotide sequence, sequence properties at the 5' and 3' termini, genome organization, morphology of the virus particle and phylogenetic analysis, PdPV-pa was confirmed as a new member of the genus *Gammapartitivirus*, family *Partitiviridae*. PdPV-pa shows closest similarity with PsV-S within *Gammapartitivirus*. The branch supports of over 90% in posterior probability in the RdRp and 100% in the CP Bayesian trees separating PdPV-pa from PsV-S (Fig 4A & 4B) and *Gammapartitivirus* species delimitation criteria (≤ 90% aa-sequence identity in RdRp and/or ≤ 80% aa-sequence identity in CP [26]) confirmed PdPV's taxonomic placement into a distinct species [25].

The occurrence of PdPV-pa infection in Pd isolates from diverse geographical locations and time suggests PdPV-pa is widely spread in North America. We could not rule out the possibility of PdPV-pa incidence in Europe considering the sample size of 17 isolates that we examined in this study. Previously, Warneke et al. [14] reported a Pd isolate from Germany (MmyotGER2) showing similar mortality effects to North American isolates when inoculated onto North American little brown bat (*M*. *lucifugus*) under experimental conditions. Unfortunately, we were not able to obtain the German isolate to evaluate the presence of PdPV-pa. However the close association of PdPV-pa in a diverse subset of the North American population of Pd sampled (35 isolates from 7 states) may provide some indications of the roles of PdPV-pa in WNS. Many mycoviruses have been reported to elicit phenotypic changes, including both hypovirulence and hypervirulence in their fungal hosts [18]. For example, the presence of *Helminthosporium victoriae 145S virus* (chrysovirus) in the plant pathogenic fungus, *Helminthosporium victoria* increased virulence in oat plants. The viral dsRNAs up-regulated *Hv-p68*, an alcohol oxidase/RNA-binding protein in the fungus that is likely responsible for the disease development [27]. Similarly, a high level of virulence was reported in the presence of a six kbp mycoviral dsRNA in *Nectria radicicola*, the causal fungus of ginger root rot [28]. The opportunistic fungal pathogen, *Aspergillus fumigatus* causing lung disease in immunocompromised humans and animals also exhibited hypervirulence in the presence of the uncharacterized A78 mycovirus [29]. We have not explored the roles of PdPV-pa in WNS in the present study, but some indirect evidence, including the difficulties in curing the fungus of PdPV-pa, the stability of the virus after numerous generations of laboratory cultures, the changes in pigmentation and the significantly reduced production of conidia in the virus-free isolate indicate close biological relationships between the fungus and the virus; hence future investigation on potential biological effects of PdPV-pa will be important.

In our attempts to cure PdPV-pa, PEG-induced stress on the matric potential was found effective. PEG being non-toxic and metabolically inert to fungi is an ideal compound to manipulate matric-induced water stress in media [30]. Matric potential influences water availability of substrates through capillary actions and particle adsorptive forces [31]. Raudabaugh & Miller [32] showed that Pd is sensitive to matric induced water stress beyond -5MPa, which is consistent with our results. In addition to the Pd growth response, normal growth at lower matric stress and significant growth inhibition as negative values of matric potential increases are characteristic of most soil fungi [32, 33]. It is possible that Pd may have originated as a soil fungus and the adaptive pressure due to competition expanded its niche. The capacity of a human pathogenic fungus, *Cryptococccus neoformans*, to infect several animals including cats, dogs, dolphins, sheep and many birds was explained based on the environmental selective pressures imposed on it while surviving in its primary niche: soil [34]. The recent findings that Pd is capable of surviving on various substrates like harvestmen, fungus gnats, moss, and cave soils in addition to bat skin [32, 35, 36], support this argument. Whether or not Pd susceptibility to matric stress is related to its origin, the inhibitory effect of the matric stress on both Pd and PdPV-pa confirms parallel biological response of both the virus and the fungus.

The genetic variation in the RdRp (<1%) and the CP (2–3%) of North American populations of PdPV-pa seems low, but in fact is quite high for partitiviruses. In studies with plant partitiviruses we find less than 1% divergence after extended periods of evolution (MR, personal observation). This higher level of variation implies a recent introduction of PdPV-pa. According to our results, only one species of this virus appears to occur in the North American isolates of Pd. The phylogenetic analysis based on a Bayesian algorithm of CP nucleotide sequences showed geographical clustering of 45 North American isolates into two main clades: USA and Canada. This indicates the diversification of PdPV-pa isolates is the outcome of geographical separation followed by sequence variation. No bat host specialization was observed. This finding is consistence with the clonal populations of Pd reported previously [10, 11] with only one mating type [12] despite its infection in several species of bats in North America.

The phylogenetic signatures of PdPV-pa isolates relating to geography provide valuable insights on the spread of WNS. The phylogeny supports two major clusters and many sub-clusters corresponding to US States of PdPV-pa isolation, suggesting connections among North American isolates, which is valuable in tracing WNS. Additionally, clustering of Pd isolates based on location was observed in several occasions within the USA clades followed by divergence, most likely for local adaptation. This analysis can be successfully expanded incorporating CP sequences of PdPV-pa from wider geographical locations to study the spread of WNS.

## Materials and Methods

### Fungal isolation and culture

*Pseudogymnoascus destructans* (Pd) was isolated from diseased bat wing tissue, live bat wing punches (2-5mm diameter) or wing swabs, cultured on 0.5X (7.5 g/L) Sabouraud dextrose agar (SDA) plates with 20 μg/ml of ampicillin, streptomycin and tetracycline at 10° C for 3 weeks in the dark. Identification of Pd was confirmed based on the species morphological characters i.e., the presence of curved conidia [1] and DNA sequences from conserved regions: internal transcribed spacer1 (ITS1), elongation factor 1α (EF-1α) and glyceraldehyde 3-phosphate dehydrogenase (gdp) genes. The pure cultures of Pd were obtained either by single spore isolation or hyphal tip cultures. For single spore cultures, actively growing Pd plates (100 mm X 15 mm) of over three weeks old were flooded with 2 ml of sterile water and gently swirled to release the spores (conidia). The spore suspension was vortexed for one minute to avoid clumping of spores. The spore suspension was then picked using an inoculating loop and spread over water agar plate (19 g/L). About 1 ml of sterile water was added in the process to help to spread the spores uniformly. The plate was viewed under a dissecting microscope and concentration of the spore suspension was adjusted so that each plate had 20–30 spores. The plate was then cultured at 7°-10°C in the dark and checked for germination every alternate day. Once the spores germinated, an agar plug was cut containing hyphae from the single germinating spore without damaging growing hyphae and then plated on a regular SDA plate to culture. For hyphal tip culture, we used the protocols described by Kanematsu *et*. *al*. [37] with some modification. We plated spore suspension on regular SDA plates as described above but when spores geminated and mycelia mats were formed they were gently overlaid with sterile Whatman cellulose filter paper soaked in SDB. The plates were then cultured for an additional two weeks until the fungal hyphae penetrated the filter paper and started growing on the upper surface. At that point the filter paper was removed and its upper surface was scraped gently and hyphal segments were suspended in sterile water. The method produced hyphal segments ranging from 4–8 cells in length that were appropriate for the hyphal tip culture. The hyphal segment suspension was then plated on SDA plates adjusting the concentration so that each plate had uniform distribution of 20–30 hyphal segments. Finally agar plugs grown from individual hyphal segments were cultured in separate plates to obtain a pure culture. The fungal isolates were stored in SDA plates for short-term storage at 4°C and at -80°C in the form of mycelia in 50% glycerol for long-term storage. All Pd isolates from Pennsylvania, one from Vermont and one from Indiana used in this study were isolated and cultured in our laboratory. The substrates (bat wings, wing punches, swabs) for these cultures were obtained from routine surveys of the Pennsylvania Game Commission (http://www.pgc.pa.gov/Wildlife/Wildlife-RelatedDiseases/WhiteNoseSyndrome). The isolates from New York, West Virginia, North Carolina, Ohio, the remaining two isolates from Vermont and all European isolates were obtained as sub-cultures from the Center for Forest Mycology Research, United States Forest Service, Madison, WI (http://www.fpl.fs.fed.us/research/centers/mycology/culture-collection.shtml). The Canadian isolates were obtained as sub-cultures from New Brunswick Museum collections, New Brunswick, Canada (http://www.nbm-mnb.ca). In addition, we obtained five isolates of *Geomyces* sp. collected from Antarctic soil from Dr. Robert A. Blanchette’s collection at the University of Minnesota and we used six isolates of *Pseudogymnoascus* sp. from cave soil in Pennsylvania for this study.

### Double-stranded RNA (dsRNA) extraction

We extracted dsRNAs from lyophilized mycelia of Pd with a minor modification in the protocol described by Márquez *et*.*al*. [38], specifically Pd was cultured using mycelial plugs or spores in 150 ml of 0.5X Sabouraud dextrose broth (SDB) supplemented with 20 μg/ml of ampicillin, streptomycin and tetracycline in a shaker at 10°C under dark conditions for three weeks prior to lyophilization. In addition to binding to CF11 cellulose (Whatman) in the presence of ethanol, the chemical nature of the dsRNA was confirmed by its resistance to DNase and RNase with NaCl concentration > 0.3M.

### Complementary DNA (cDNA) synthesis and cloning

Approximately 2 μg of dsRNA were mixed with 2 μM of random primer-dN10 with a linker sequence (5'CCTTCGGATCCTCCN_10_3'), 0.5 mM of Tris-EDTA (pH 8.0) and nuclease-free water to a final volume of 12 μl, and boiled for 2 min. The mixture was incubated on ice, and 8 μl of Reverse Transcriptase (RT) mix (SuperScript^TM^ III RT 200U, 5X First-Strand buffer 4 μl, 0.1M DTT 1 μl and dNTP 0.5 mM as recommended by the manufacturer) were added and incubation continued at 50°C for 1.5 hours. The newly synthesized cDNA mixture was then incubated with 10 μg of boiled RNase A (Sigma) for 15 min. at room temperature and cleaned with E.Z.N.A Cycle Pure Kit (Omega Bio-tech) according to the manufactures instruction. About 0.5 μg of cleaned cDNA was used as a template for a 25 μl polymerase chain reaction (PCR) with *Taq* DNA Polymerase (ThermoFisher Scientific), buffers, dNTPs supplied with 1μM concentration of the primer (5'CCTTCGGATCCTCC 3'). The PCR was completed in a Idaho Technologies Rapid Cycler with a slope setting of 5, using the following cycles: 1 cycle of 94°C, 1 min.; 25 cycles of 94°C, 0 sec., 45°C, 0 sec., and 72°C, 15 sec.; 1cycle of 72°C, 5 min.; 1 cycle of 37°C, 5 min. The PCR product was cleaned and cloned into the pGEM-T Easy Vector System (Promega) according to the manufacturers instructions. Sequence analysis of the cDNA plasmid clones were done by the Genomic Core Facility of Pennsylvania State University, University Park, PA. The sequences obtained were trimmed for plasmid and primer sequences and assembled using de *novo* assembly in Geneious version 8.0.2 [39]. All cloning and sequence analysis was based on the dsRNA from the LB-01 isolate cultured from a little brown bat from Pennsylvania.

### Terminal sequencing

RNA ligase mediated-rapid amplification of cDNA ends (RLM-RACE) was performed to determine the terminal sequences of the PdPV-pa dsRNA segments. A 5'-phosphorylated oligodeoxynucletide (5'-PO_4_-GGAGGATCCGAATTCAGG 3') was ligated to the dsRNA termini as an adaptor before synthesizing cDNAs using a complementary primer (5'CCTGAATTCGGATCCTCC3') in combination with the internal primers designed for PdPV-pa RNA1 and RNA2 (RNA 1: 5'TTCAAGTTCGCCCTGTACC3'F, 5'TGAGCGAATGGAAGGTTG3'R; RNA 2: 5'CGCGTAATCATGACGACC3'F, 5'CCGAGGAGCACACACTATC3'R) in RLM-RACE. Ligation reactions were done in 50% PEG with 2 U of T4 RNA ligase 2 (New England BioLabs) mixed with approximately 2 μg of dsRNA along with the primers mentioned above and buffer supplied according to the manufacturers instructions, and incubated at 4°C overnight. RT-PCR of the primer-ligated dsRNA was performed exactly like described in the cDNA synthesis above except the enzyme used was Avian Myeloblastosis Virus (AMV) RT (New England BioLabs). The amplicons were cloned followed by sequence determination using Sanger sequencing. The complete nucleotide sequences of PdPV-pa RNA 1 and PdPV-pa RNA 2 have been deposited in GenBank with accession numbers KY20754 and KY207544, respectively.

### Sequence analysis

Consensus sequences for PdPV-pa RNA 1 and RNA 2 were analyzed for the open reading frames (ORFs) using ORF finding operation in Geneious version 8.0.2. A sequence similarity search was conducted with BLASTn and BLASTx available online from the National Center for Biotechnology Information (NCBI).

### Northern blot analysis

Northern blotting was performed using non-radioactive isotopes probes, digoxigenin (DIG)-11-dUTP-labeled DNA fragments according to the manufacturers instructions (Roche Diagnostics). Representative clones of PdPV-pa RNA 1 and RNA 2 in the range of 500–700 bp were selected and the labeling was done in a PCR with DIG-11-dUTP and dNTPs mix (DIG-11-dUTP:dTTP = 1:3; with equimolar amount of dATP, dCTP and dGTP), *Taq* DNA Polymerase (ThermoFisher Scientific), specific primers and buffer in Idaho Technologies Rapid Cycler as described above. About 2 μg of PdPV-pa dsRNA was electrophoresed in 1.2% agarose gels and subsequently denatured by saturating with freshly prepared 50mM NaOH for 30 min followed by neutralization in 50mM sodium borate for 5 min. The cycle was repeated three times before dsRNA was transferred to a nylon membrane (Hybond N+ Amersham) by capillary action overnight. The membranes were UV-cross-linked in a Stratalinker at 200 J. Hybridization and washings were carried out as described by Li et al. [40] except we performed prehybridization and hybridization at 52°C instead of 42°C. The blots were incubated in antibody solution, anti-DIG-AP Conjugate (Roche) and CDP-STAR (Roche) for chemiluminescence detection.

### Virus purification

Virus particles were purified following methods described by Sanderlin and Ghabrial [41] with some modifications. Eight g of lyophilized mycelia of Pd isolate BB-06 was ground to powder in the presence of liquid nitrogen. The homogenates were mixed with extraction buffer (0.1 M sodium phosphate. pH 7.6 containing 0.5% (v/v) thioglycolic acid) and mixed with chloroform followed by low speed centrifugation at 7000 rpm for 15 min at 4°C. The virus containing supernatant was then subjected to two cycles of differential centrifugations (low speed at 7000 rpm for 15 min and ultracentrifuge at 35,000 for 1.5 hours). During the ultracentrifuge cycle, the virus containing supernatant was underlaid with a 10% sucrose cushion. The final pellets were suspended in 1 ml of 0.03 M sodium phosphate buffer pH 7.6.

The virus preparation was examined under JEOL 1400 transmission electron microscope after negatively staining with uranyl formate in the Microscopy and Imaging Facility at Penn State College of Medicine, Hersey, PA.

### Curing the fungus

For the heat stress, actively growing Pd plates in three replicates were exposed to room temperature (22–23°C), 37°C and 42°C for 2, 6, 12 and 24 hours before culturing the mycelia plugs from each treatments in liquid medium (SDB) under normal laboratory culture conditions for Pd described above. During the treatments, Pd plates in three replicates were also grown under normal culture condition as controls. The fungal mycelia were then harvested after three weeks to extract dsRNAs. However, only samples treated at room temperature and 37°C for 2 hours grew. Single spore isolation and hyphal tip cultures were done as described under the section, fungal isolation and culture.

The protoplast isolation from Pd was performed on mycelia (~ 1.7 g) harvested from SDB culture after two weeks at 10°C in a shaker (200 rpm) in the dark. The fungal mycelia were collected by centrifugation at 90 × g for 5 min followed by washing with KCl buffer (0.6 M, pH 5.8) as an osmotic stabilizing agent. The mycelia was treated with lysing enzyme mixture (Lysing enzyme from *Trichoderma harzianum* 20 mg/ml and driselase 20 mg/ml from Sigma) prepared in KCl buffer and incubated at 10°C at 70 rpm in the dark. Protoplast production was checked every half an hour until 35–40 protoplasts were observed under a 40X field with 10 μl of the mixture. The mixture was then passed through double-layered miracloth (VWR) soaked in STC buffer (1.2 M Sorbitol; 10 mM Tris-HCl, pH 7.5; 20 mM CaCl_2_) to filter out the cell debris. The filtrate was centrifuged at 90 × g for 5 min to collect the protoplasts which were resuspended in regeneration media (0.5% yeast extract, 2% glucose, 0.6 M Sorbitol and 25 mM CaCl_2_) followed by incubation at 10°C at 70 rpm in the dark. Once the protoplasts recovered completely with cell wall growth, they were transferred to agar supplemented regeneration media (0.5% yeast extract, 2% glucose, 20% sucrose and 1% agar) and the concentration adjusted so that each plate had 25–30 uniformly distributed cells. The plates were then incubated under normal culture condition for Pd until hyphae developed uniformly around each protoplast without touching each other. Individual colonies were then picked and cultured in SDA.

We also treated Pd with the antiviral drugs cycloheximide and ribavirin at different concentrations in SDA media. Cycloheximide was used at 2 μg/ml, 5 μg/ml, 10 μg/ml, 15 μg/ml and 25 μg/ml concentrations. Ribavirin treatment was at 80 μM, 100 μM, 150 μM, 200 μM and 300 μM concentrations. Three passages with both cycloheximide and ribavirin were also performed with higher concentrations.

For PEG induced matric stress on water availability we used PEG 8000 (Fisher BioRegents) in a modified Spezieller Nährstoffarmer liquid media (SN: 0.02 g sucrose, 0.02 g glucose, 0.08 g KNo_3_, 0.08 g KH_2_Po_4_, 0.04 g MgSo_4_.7H_2_O and 0.04 g NaCl/L) to make media with water potential gradients of -1 MPa, -2 MPa, -3MPa, -4 MPa, -5 MPa and -6 MPa. The amount of PEG 8000 in gram/gram of water was calculated based on Michel [42] equation: Ψ (water potential) = 1.29 [PEG]^2^T – 140[PEG]^2^–4 [PEG] and the value was adjusted to the Pd culture temperature of 10°C. An agar plug containing actively growing Pd was placed in 50 ml autoclaved modified SN liquid media with a targeted amount of PEG 8000 (-1 MPa: ~ 0.075 PEG g/g of water, -2 MPa: ~ 0.11 PEG g/g of water, -3 MPa: ~ 0.14 PEG g/g of water, -4 MPa: ~ 0.16 PEG g/g of water, -5 MPa: ~ 0.19 PEG g/g of water and -6 MPa: ~ 0.21 PEG g/g of water) and grown as described above. After three weeks, pieces of newly growing mycelia of Pd were transferred to normal SBD routinely used to culture Pd and the fungus was harvested after a normal culture period. The fungi from different treatments were examined for PdPV-pa both by dsRNAs gel electrophoresis and RT-PCR with PdPV-pa specific primers. In all methods Pd isolate LB-01 was used.

### Diversity and phylogenetic analysis of PdPV

Genetic variation in North American PdPV-pa isolates were determined by sequence analysis of RdRp and CP segments amplified in RT-PCR using specific primers. The primer pairs specific to RdRp (5'ATGGAAGTATCTCCTTTCG3'F, 5'GTATAGAAGATTGAGTGCC3' R) and CP (5'ACTCTGTGTTAACGGAGG3'F, 5'CTGTAGTTGACACCTGTACC3'R) were designed from the consensus sequences of RNA 1 and RNA 2 assembled from LB-01 isolate cloned sequences. PCR products using RdRp and CP specific primers from 45 North American PdPV isolates were sequenced and aligned with MUSCLE default settings in the program Geneious 8.0 [39]. The RdRp sequences have been deposited in GenBank under accession numbers KY207498 to KY207552 and the CP sequences have been deposited in GenBank under accession numbers KY207453 to KY207497. The alignment was visually corrected as necessary before recording segregating and singleton sites. The average percentage identity for each sequence was calculated by taking the average from a pairwise percentage identity matrix generated from the sequence alignment. Phylogenetic analysis was performed using MrBayes [43] implemented via a plug-in in Geneious. The amino acid sequences were used in studying the evolutionary relationships of PdPV-pa within the genus *Gammapartitivirus*. The tree was constructed using amino acid sequence (RdRp and CP) of 10 approved species of *Gammapartitivirus* available in the GenBank. The sequences of *Pepper cryptic virus 1*, type member of genus *Deltapartitivirus*, which is the closest group to *Gammapartitivirus* in *Partitiviridae* family was used as outgroup. We used nucleotide sequences (CP) to study phylogenetic relationships of PdPV-pa in North American population. The nucleotide sequence of PsV-S CP was used as outgroup in the analysis. In Bayesian trees construction using amino acid sequence of the RdRp and CP ORFs, Jukes-Cantor substitution model was applied and for nucleotide sequences of CP General time-reversible (GTR) model with gamma rate variation was used based on the best model tested out of 28 models.

## Supporting Information

S1 Table Accession numbers for sequences from Genbank used in the phylogenetic placement of PdPV-pa in the *Gammapartitivirus* genus. (DOCX)

S2 Table Analysis of virus status of related fungal species. (DOCX)

S1 Appendix Analysis of detection limits of PdPV-pa by RT-PCR (DOCX)
