# Supplementary material for: Using a Novel Partitivirus in Pseudogymnoascus destructans to Understand the Epidemiology of White-Nose Syndrome
Source: PLoS Pathog. 2016 Dec 27;12(12):e1006076. doi: 10.1371/journal.ppat.1006076 (PMC5189944; doi:10.1371/journal.ppat.1006076)
Supplement: S1 Table — (DOCX) [file ppat.1006076.s001.docx]

**Supporting Information Table S1. GenBank accession number for RdRp and CP sequences used in phylogenetic analyses with PdPV**

| Partitivirus species | Accession No. for RdRp | Accession No. for CP |
| --- | --- | --- |
| *Aspergillus fumigatus partitivirus* 1 | CAY25801.2 | CAZ61323.2 |
| *Aspergillus ochraceous virus* | ABC86749.1 | ABV30676.1 |
| *Botryotinia fluckeliana partitivirus 1* | YP_001686789 | YP_001686790 |
| *Discula destructiva virus* 1 | NP_116716 | NP_116742 |
| *Fusarium solani virus* 1 | NP_624350 | NP_624351 |
| *Gammeniella abietina RNA virus* MS1 | NP_659027 | NP_659028 |
| *Ophiostoma partitivirus* 1 | CAJ31886 | CAJ31887 |
| *Penicillium stoloniferum virus S* | YP_052856 | YP_052857 |
| *Ustilaginoidea virens partitivirus* 1 | AGO04402 | AGO04403 |
| *Verticillium dahlia partitivirus* 1 | YP_009164038 | YP_009164039 |
| *Pepper cryptic virus* 1 | AEJ07890 | AEJ07891 |
