# Supplementary material for: Using a Novel Partitivirus in Pseudogymnoascus destructans to Understand the Epidemiology of White-Nose Syndrome
Source: PLoS Pathog. 2016 Dec 27;12(12):e1006076. doi: 10.1371/journal.ppat.1006076 (PMC5189944; doi:10.1371/journal.ppat.1006076)
Supplement: S2 Table — (DOCX) [file ppat.1006076.s002.docx]

**Supporting Information Table S2: Cultures of taxa closely related to *Pseudogymnoascus destructans* tested for PdPV-pa**

| **Culture ID** | **Taxa** | **Location** | **PdPV-pa^1^** |
| --- | --- | --- | --- |
| LB-RT-G.vana | *Gymnoascoides vinaceous* | PA, USA | - |
| LB-RT-Gymno | *Gymnoascoides sp.* | PA, USA | - |
| LB-RT-Myxo | *Myxotrichum sp.* | PA, USA | - |
| P_sd_ | *Pseudogymnoascus sp* | Canoe Creek, PA, USA | - |
| P_s2_ | *Pseudogymnoascus sp.* | Canoe Creek, PA, USA | - |
| P_s4_ | *Pseudogymnoascus sp.* | Canoe Creek, PA, USA | - |
| P_s5_ | *Pseudogymnoascus sp.* | Canoe Creek, PA, USA | - |
| DC7N-2 | *Geomyces sp.* | Antarctica: Ross Island | - |
| 5S9-4 | *Geomyces sp.* | Antarctica: Antarctic Peninsula | - |
| 5NP11-3 | *Geomyces sp.* | Antarctica: Antarctic Peninsula | - |
| 5NB8-2 | *Geomyces sp.* | Antarctica: Antarctic Peninsula | - |
|  |  |  |  |
|  |  |  |  |

^1^ ‘-’ indicates absence of PdPV-pa when tested by dsRNA extraction and RT-PCR using specific primers for the RdRp of PdPV-pa.

**Note:** Pennsylvania cultures were collected by Dr. Barrie E. Overton, Lock Haven University, PA, and Antarctic cultures were received from Dr. Robert A. Blanchette, University of MN.
