# Supplementary material for: Using a Novel Partitivirus in Pseudogymnoascus destructans to Understand the Epidemiology of White-Nose Syndrome
Source: PLoS Pathog. 2016 Dec 27;12(12):e1006076. doi: 10.1371/journal.ppat.1006076 (PMC5189944; doi:10.1371/journal.ppat.1006076)
Supplement: S1 Appendix — (DOCX) [file ppat.1006076.s003.docx]

**Supporting Information Appendix S1 PdPV-1 detection limit measurement**

To assess the PdPV-pa detection limit, we extracted total nucleic acid followed by dsRNA enrichment using a conidia suspension collected from wild type isolate of BB06 infected with PdPV-pa. The number of conidia in the suspension was estimated by counting spores using a hemocytometer. We also extracted dsRNA from approximately same amount of mycelia to conidial mass of BB06 wild type isolate as a control (Fig 1.). The concentration of dsRNA extracted from conidia was determined using a spectrophotometer. Then, we diluted the dsRNA extracted from conidia by serial dilution factors of 5, 10, 15 and 20 and performed RT-PCR as described in the material and method section using RdRp specific primer of PdPV-1 (Fig 2.).


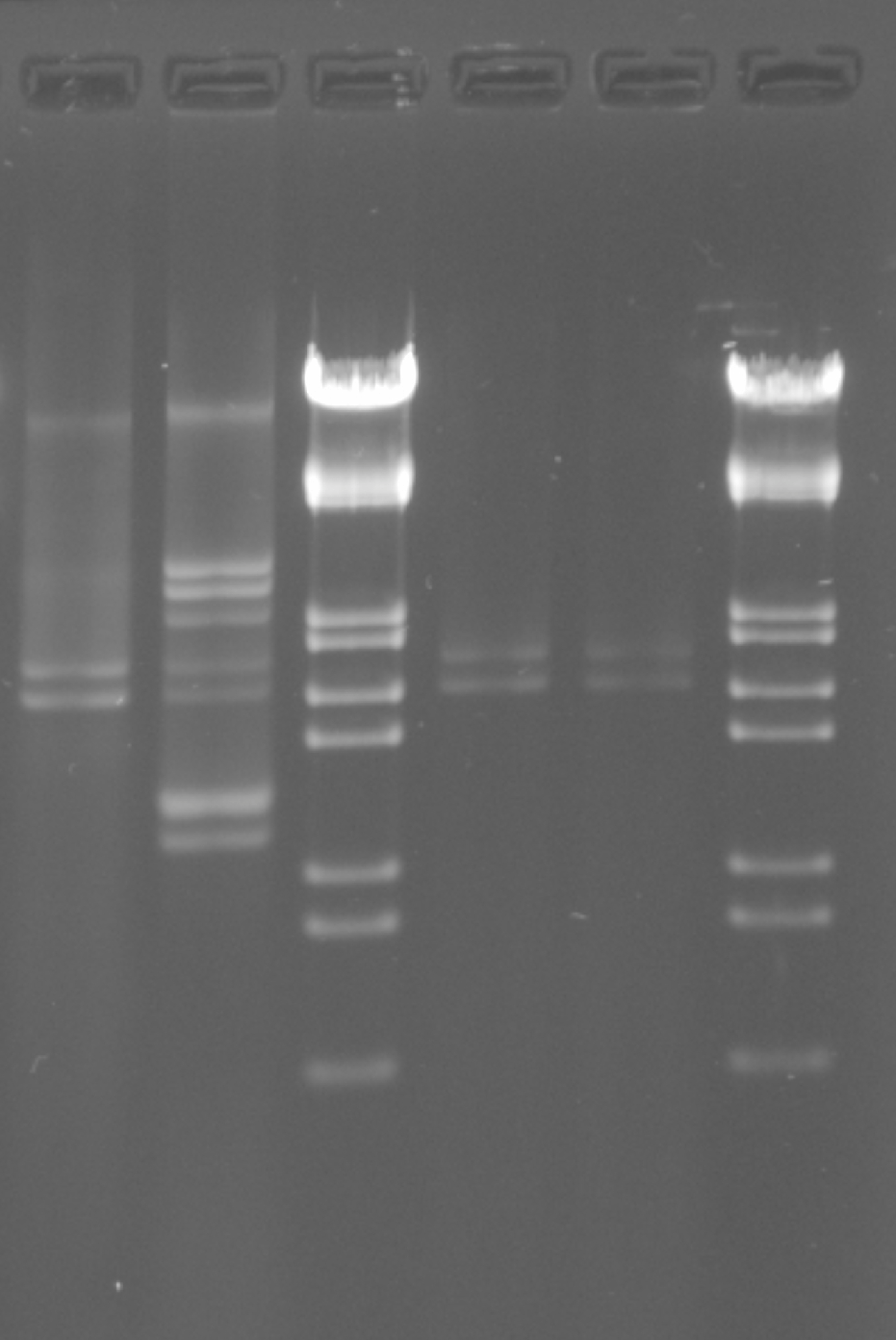


M BB06 BB06’


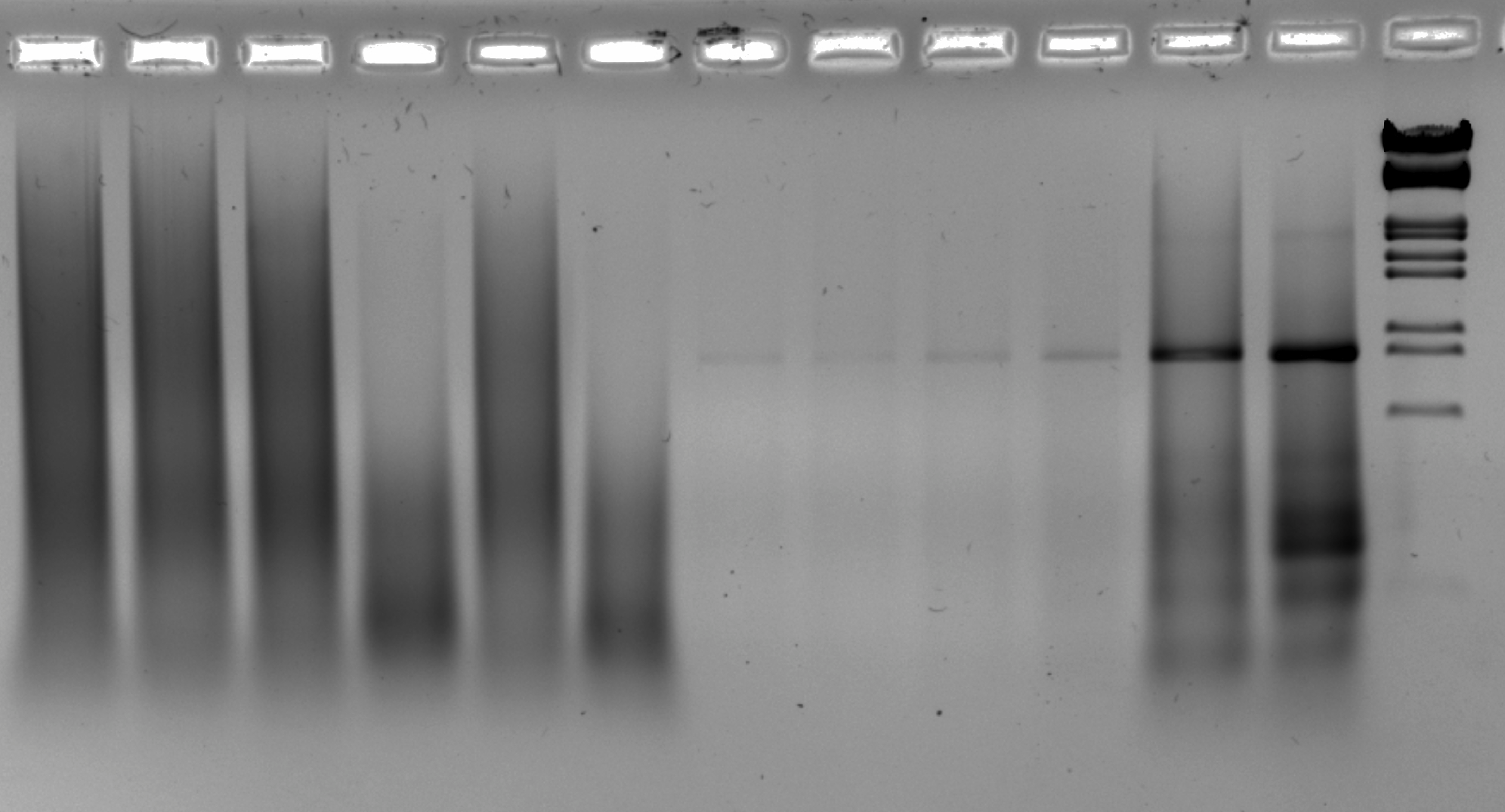


M LB01 BB06 BB06’ X 5 X 10 X 15 X 20

Fig 1. dsRNA extracted from Fig 2. Amplicons from RT-PCR using

BB06 mycelia (BB06) and BB06 PdPV-1 specific RdRp primers using

conidia (BB06’). dsRNA template from BB06 conidia

(BB06’) and its dilutions at X5, X10,

X15 and X20 factors. LB01 and BB06

were two controls used with dsRNAs

extracted from mycelia.

Calculation of PdPV-1 detection limit

dsRNA concentration measured by spectophotometer = 6.156 μg/ml

Number of conidia in 1μl of suspension = 562,980 = 562,980,000/ml

PdPV-1, RdRp = 1761 bp

Molar mass of PdPV-1, RdRp = 1.14465E+12 μg (considering 1 mole of 1bp = 650 g)

Number of moles in 6.156 μg of dsRNA = 6.156/1.14465E+12 = 5.37806E-12

Number of molecules = 5.37806E-12*6.022*10^23 = 3.23867E+12

Copy number of PdPV-1, RdRp/conidia = 3.23867E+12/562980000 = 5752.726

Copy number of PdPV-1/conidia at X15 dilution (detection limit) = 5752.726/15 = 383.5
